# Improvement of Foaming Characteristics and Stability of Sterilized Liquid Egg with Egg White Hydrolysate (EWH)

**DOI:** 10.3390/foods10061326

**Published:** 2021-06-09

**Authors:** Hen-Yo Ho, Jhih-Ying Ciou, Yi-Ting Qiu, Shu-Ling Hsieh, Ming-Kuei Shih, Min-Hung Chen, Chao-Wen Tu, Chang-Wei Hsieh, Chih-Yao Hou

**Affiliations:** 1 Department of Seafood Science, National Kaohsiung University of Science and Technology, Kaohsiung City 811, Taiwan; areyou1720@gmail.com (H.-Y.H.); C107176201@nkust.edu.tw (Y.-T.Q.); slhsieh@nkust.edu.tw (S.-L.H.); e23magic@gmail.com (C.-W.T.); 2 Department of Food Science, Tunghai University, Taichung City 407, Taiwan; jyciou@thu.edu.tw; 3 Graduate Institute of Food Culture and Innovation, National Kaohsiung University of Hospitality and Tourism, 812301 No. 1, Songhe Rd., Xiaogang Dist., Kaohsiung City 811, Taiwan; mkshih@mail.nkuht.edu.tw; 4 Agriculture & Food Agency Council of Agriculture Executive, Yuan Marketing & Processing Division, 54044 No. 8, Kuang-Hua Rd., Chung-Hsing New Village, Nantou City 540, Taiwan; cmh@mail.afa.gov.tw; 5 Department of Food Science and Biotechnology, National Chung Hsing University, 145 Xingda Rd., South Dist., Taichung City 402, Taiwan; 6 Department of Medical Research, China Medical University Hospital, Taichung City 404, Taiwan

**Keywords:** liquid egg, egg white hydrolysate, foaming characteristics, stability

## Abstract

A pasteurized liquid egg leads to protein denaturation and degradation of processing properties, whereas non-pasteurized eggs may have food safety risks. If the negative impact of the pasteurization process on liquid eggs can be reduced, for example, the loss of stability and foamability, companies will be willing to purchase pasteurized eggs, thereby reducing food safety risks. Therefore, in this study, specific hydrolyzation conditions were used to produce egg white hydrolysate (EWH) with a lower molecular mass of amino acid and peptide fragments, and the effects of various concentration of EWH refilling on pasteurized liquid egg properties were investigated. The results showed that up to 30.1% of EWH was hydrolyzed by protease A and papain. Adding 1% (*w*/*w*) EWH can improve the negative charge potential value, surface tension, viscosity, and weight loss analysis of the sample. In addition, the cake structure and the appearance was acceptable to consumers. Therefore, to ensure its efficient use in the baking industry and considering the cost and stability, 1% (*w*/*w*) EWH was chosen as the best concentration.

## 1. Introduction

In Taiwan, the production technology and market need for fresh chicken eggs expanded recently, promoting sales in the egg industry and turning many sideline businesses models into large-scale businesses. Chicken eggs were mainly sold as whole eggs. However, as industries grew, liquid eggs were made to save the time taken to break those eggs, which are most popular in the baking industries for cake and bread production, and to reduce bakery waste in the kitchen.

*Salmonella* contamination is one of the most serious food safety risks for chicken eggs. *Salmonella* contamination causes food poisoning, which is a global public health threat and one of the most common infectious diseases in humans and animals [1]. Based on the statistics of Taiwan’s Food and Drug Administration, the occurrence of egg food safety incidents is frequent and leads to consumer panic. Therefore, many industries take care regarding such issues and try to reduce the contamination of *Salmonella* or other harmful microorganisms in sterilized eggs before use. Most eggs are sterilized by pasteurization; however, the sterilization process may cause protein denaturation [2]. Although pasteurization can reduce contamination by *Salmonella* or other harmful microorganisms, if the duration is too long or the temperature is too high, proteins will denature and destroy the processing properties, including the foaming properties of egg whites [3,4] and stability of whipped egg whites [5,6]. This is because the coagulation temperature of egg white is 57 °C, whereas that of egg yolk is 65 °C [7,8]. If this occurs, the bakery industry would not be able to work on their daily production. Hence, fresh whole eggs or non-sterilized eggs are used, owing to the limitation of sterilized eggs.

Recently, many studies on the negative effects of pasteurization have been published, such as those on the foaming and air–water interfacial characteristics of solutions containing both gluten hydrolysate and egg white protein [9], the effect of Persian gum and Xanthan gum on the properties and stability of pasteurized fresh egg white foam [10], the combination of egg white protein and microgels to stabilize foams, and the effects of processing treatments [11].

According to these research articles, egg white hydrolysate (EWH) can be used to enrich preprocessed food, increase nutrition, improve food texture, and enhance forming properties, gelatinization, and flavor [12]. Therefore, this study aimed to expand the limited use of sterilized liquid eggs in the bakery industry. The effects of pasteurization on the processing properties of liquid eggs were negated by hydrolyzation. Fresh egg whites were diluted and subjected to enzymatic hydrolyzation to release lower-molecular-mass substances (e.g., peptides and amino acids), and pasteurization was used to destroy possible pathogens. The hydrolyzed egg whites were then refilled with sterilized liquid eggs to counteract the negative effects of pasteurization. This solved contamination by *Salmonella* and other microorganisms and simultaneously ensured the quality of eggs and food safety. The conditions for stability, foaming properties of sterilized egg white, and application in the bakery industry were evaluated to enhance purchasing intention and provide safe products for consumers.

## 2. Materials and Methods

### 2.1. Materials

Non-sterilized liquid eggs were purchased from Sheng Da Foods Co., Ltd. (Kaohsiung, Taiwan). Sterilized liquid eggs were purchased from Jin Ding Foods Co., Ltd. (Kaohsiung, Taiwan). Protease A was purchased from Ho Jun Biotechnology Co., Ltd. (Taoyuan, Taiwan). Papain was purchased from Champion Co., Ltd. (Taipei, Taiwan). Bovine serum albumin (BSA) and Reagents A, B, and S were purchased from Bio Rad, represented by Genmall Biotechnology Co., Ltd. (Taipei, Taiwan). Serine was purchased from Sigma-Aldrich, represented by Taiwan Merck Co., Ltd. (Taipei, Taiwan). Sodium tetraborate decahydrate (borax) was purchased from Showa Chemical Industry Co., Ltd. (Tokyo, Japan). Ethanol (95%), sodium dodecyl sulfate (SDS), and ortho-phthalaldehyde (OPA) were purchased from Alfa Aesar, represented by Echo Chemical Co., Ltd. (Taipei, Taiwan). Dithiothreitol (DTT) was purchased from R&D Systems, represented by Union Biomed Inc. Co., Ltd. (Taipei, Taiwan).

### 2.2. Egg White Hydrolysate Preparation

Fresh egg white was diluted with water to 12.5% and homogenized. Then, the diluted solution was heated to 50 °C and maintained in a thermostatic water bath. Next, 0.047% enzyme (protease A and papain at a ratio of 1:1) was prepared by dissolving the enzyme with five times the amount of deionized water and adding it to the diluted solution prepared earlier for the two-stage hydrolyzation. Hydrolyzation was performed at 50 °C for 1 h and again at 68 °C for 2 h. Then, the temperature was maintained for 10 min at 95–98 °C to deactivate enzymatic activity. The diluted solution was then centrifuged (6000× *g*) for 5 min at 4 °C, and the supernatant was withdrawn and pasteurized for 3.5 min at 58 °C. EWH was prepared and stored at 7 °C until analysis.

#### 2.2.1. Protein Content Analysis

In accordance with the method of [13], standard solutions of BSA with concentrations of 1600, 800, 400, 200, and 100 ppm were used. EWH was diluted 50 times for analysis. The standard solutions and EWH were placed at 5 μL/well in a 96-well plate, and 25 μL/well of Reagent A and Reagent B (50:1) (mixed well before addition) and 200 μL/well of Reagent S were added. The sample was placed in the dark and reacted under shaking conditions for 15 min, and the absorbance was determined at 750 nm using an ELISA reader. The protein content (%) of EWH was then calculated.

#### 2.2.2. Analysis of Degree of Hydrolysis (DH)

In accordance with the method of [13], 100 ppm serine was used as the standard solution, and EWH was prepared with 0.08% protein content. Then, 7.620 g borax and 200 mg SDS were dissolved in 150 mL distilled water; following this, 4 mL of 95% ethanol, 160 mg OPA, and 176 mg DTT were added, and the solution was made up to 200 mL to prepare the OPA reagent. Subsequently, 20 μL/well sample and 150 μL/well OPA reagent were reacted, and the absorbance was determined at 340 nm. The DH was determined using the following formula:

DH (%) = [(OD_Sample_ − OD_Blank_)/ (OD_Standard_ − OD_Sample_) × (0.9516 × 0.1)/ (X × P) − β]/ (α × h_tot_) × 100 (1)

OD_Standard_: absorbance of standard solution (Serine)

OD_Sample_: absorbance of sample solution

OD_Blank_: absorbance of blank

X: volume of 1 mL 0.08% protein content sample (mL)

P: protein content of sample (%)

α, β, and h_tot_: 1.0, 0.4, and 8.0, respectively

### 2.3. Sample Preparation

Non-sterilized liquid egg white (NSLE group) and sterilized liquid egg white (SLE group) were purchased, and experimental groups were prepared by adding 1%, 3%, and 5% (*w*/*w*) EWH in the SLE group.

#### 2.3.1. Zeta Potential Analysis

The method is described in [10,14]. The zeta potentials (mV) of all sample groups (NSLE, SLE, and experimental groups) were determined using a zeta potential analyzer at 25 °C. A larger value of negative charge indicates better stability.

#### 2.3.2. Surface Tension Analysis

The method is described in [10,14]. The sample angles (°) of all the sample groups were determined using a tensiometer, and the surface tension was calculated using the following formula. Samples with better stability exhibit larger surface tension values.

(2) $$\mathrm{Surface}\;\mathrm{tension}\;(\mathrm{erg}/{\mathrm{cm}}^{2})=\frac{\;r_{s}\;}{\theta_{s}}=\frac{\;r\;}{\theta }$$

$r_{s}$: surface tension of water = 72 (erg/cm^2^)

$\theta_{s}$: degree of water (°)

r: surface tension of sample (erg/cm^2^)

θ: degree of sample (°)

#### 2.3.3. Viscosity Analysis

The method is described in [10,14]. The viscosity (mPa·s) of the samples was determined using a viscometer and rheometer with a shear rate of 0.1–300 s^−1^ at 25 °C. The stability increases with the viscosity.

#### 2.3.4. Color Analysis

A colorimeter was used to determine the color of the samples. The Hunter L, a, and b values represent the lightness, red–green component, and yellow–blue component of the samples, respectively. The standard illuminant B served as a representative of noon sunlight, with a correlated color temperature (CCT) of 4874 K. The ΔE represents the color difference of the samples.

(3) $$\Delta E=\sqrt{{{(L}_{2}^{*}{–L}_{1}^{*})}^{2}{+(a}_{2}^{*}{–a}_{1}^{*}{)}^{2}{+(b}_{2}^{*}{–b}_{1}^{*}{)}^{2}}$$

### 2.4. Foam Sample Preparation

The preparation was similar to that described in Section 2.3; 100 mL of each sample was whipped for 3–5 min until the largest volume was obtained.

#### 2.4.1. Drainage Analysis

The method is described in [10]. The foam of each whipped group was tested by determining the weight of the sample drained through filter paper for 30 min. The sample weight (g) was measured every 5 min. Larger weight losses indicate poorer stability.

#### 2.4.2. Physical Analysis

In accordance with the method of [10,14], the foam of all samples was analyzed with respect to the overrun (OR), air phase, and foam density by determining the volume of the sample before and after whipping. The volume was obtained by weighing 100 mL of the foam. Larger values of the OR and air phase indicate lower density and better foaming properties.

OR (%) = (V_f_ − V_1_)/V_1_ × 100 (4)

Air Phase (%) = OR/(OR + 100)(5)

(6) $$\mathrm{Foam}\;\mathrm{Density}\;(g/{\mathrm{cm}}^{3})=m_{100f}/m_{100H_{2}O}$$

where V_f_—foam volume; V_l_—initial liquid volume; $m_{100f}$—100 mL foam mass; $m_{100H_{2}O}$—100 mL water mass.

#### 2.4.3. Microstructure Analysis

In accordance with the method of [14], the microstructure of all foam samples (0.5 × 0.5 cm^2^) was observed under an inverted microscope at 50× magnification to determine the foam condition and then analyzed based on a CCD (charge coupled device) image. Larger bubble holes indicate weaker foaming ability of the egg white.

### 2.5. Baking Cake Sample

Egg white sample preparation was similar to that described in Section 2.3; 160 g egg white sample and 80 g commercial sterilized egg yolk were used in place of whole eggs (the ratio of an egg is 2:1). The prepared egg sample was beaten in a bain-marie until the temperature reached 40 °C; then, it was mixed well with 120 g fine sugar and 48 g honey and beaten in a stand mixer at a fast speed until the mixture was pale and fluffy. Thereafter, 24 g of milk at 40 °C and 120 g of sieved cake flour were added to the batter for mixing. The mixed batter was transferred to a pan and baked at 150–170 °C for 20 min; the pan was rotated halfway through and baked at 150–170 °C until a toothpick inserted into the center of the cake came out clean, approximately 40 min.

#### 2.5.1. Color Analysis

The color of the baked cake was determined using a colorimeter (Nippon Denshoku, SA-4000, Tokyo, Japan) at room temperature. The standard illuminant B served as a representative of noon sunlight, with a correlated color temperature (CCT) of 4874 K. The method of determination was similar to that in Section 2.3.4.

#### 2.5.2. Microstructure Analysis

In accordance with the method of [14], all the samples were first cut into 0.5 cm^3^ cubes, and the bubble condition was observed under an inverted microscope at 50× magnification and analyzed with a CCD image. Larger bubble holes indicate better foaming ability of the egg white.

#### 2.5.3. Cross-Sectional Height Analysis

Baked cake samples were cut in half and the rising height of the cake was observed and measured with a ruler; a greater rising height indicates better foaming properties.

#### 2.5.4. Organoleptic Analysis

The cake samples were placed in a clear plate under regular fluorescent lights. The serving temperature of the cake samples was approximately equal to room temperature, and the room temperature was ~25 °C. One piece (about 30 g) was tasted for each group, and the panelists refreshed their mouth by drinking sparkling water twice between each group. In the present study, a sensory-profile analysis with blind testing was conducted by 20 panelists (9 females and 11 males, aged between 25 and 40 years) from National Kaohsiung University of Science and Technology. The majorities of panelists were previously trained on sensory evaluation techniques and had experience of being taste testers. All cake samples were subjected to the 9-point hedonic scale test, and 20 test panels were evaluated for appearance, aroma, sweetness, bitterness, texture, and overall acceptance. The score spanned 1–9 points, where 1 represented weak (dislike), 5 represented medium (like), and 9 represented strong (very much like) acceptance.

### 2.6. Statistical Analyses

All experiments were performed at least twice, and three samples were used for each test. Data were collected and analyzed using one-way ANOVA and Duncan’s test. Differences were considered significant at *p* < 0.05. All statistical analyses were performed using SPSS (version 12.0, IBM Inc., St. Armonk, NY, USA).

## 3. Results

### 3.1. Analysis of Egg White Hydrolysate

Previous studies indicated that protein content and hydrolyzation ability were closely related to the effects discussed in subsequent experiments. In this study, the protein content of EWH was 6.19%, and the highest DH according to literature was 25% [15]; however, the DH increased to 30.11% upon the addition of protease A and papain.

### 3.2. Analysis of Stability

The zeta potential is an important and readily measurable indicator of the stability of colloidal dispersions. One of the important factors that indicate stability is a zeta potential of 15.0 mV [16], which is due to the electrostatic repulsion of electric charge on the sample surface. When the potential is small, attractive forces may exceed this repulsion, and the dispersion may break and flocculate. The aggregation ability of proteins results from covalent and non-covalent interactions, which affect the foaming properties of the whipped sample; larger values of negative charge indicate better stability [17]. However, surface tension and viscosity are also related; when a sample is whipped into foam, it forms a thin layer of protein membrane within the gas–liquid interface, and the surface tension of the sample increases, raising the viscosity [18]. Therefore, viscosity is an important factor for foam stability of a sample; higher surface tension and viscosity values indicate greater stability [19]. 

As shown in Table 1, SLE showed a lower negative charge value, surface tension, and viscosity than NSLE. This indicated that liquid eggs had lower stability after pasteurization, whereas those with different ratios of EWH were more stable. Due to the strong electrostatic repulsion between proteins, high zeta potential indicates protein stability in solution, while low zeta potential indicates weaker repulsive force and greater protein–protein aggregation tendency [20]. The low zeta potential values obtained in this study indicate that SLE is unstable and tends to aggregate. However, the addition of EWH in SLE can significantly increase the absolute value of zeta. In contrast, with the increase of EWH content in SLE, the absolute value of zeta potential becomes lower (in Table 1) (*p* < 0.05), suggesting that the zeta potential is affected by the ratio between SLE and EWH. 

Zhang and Li (2018) reported that the particle size and zeta potential of the surfactin–alkaline protease system are strongly dependent on the concentration of surfactin [21]. Combining the result of the molecular aggregation of the system with the falling zeta potential of the system at low concentration of surfactin, it can be concluded that there are electrostatic interactions between surfactin and alkaline protease molecules at low concentrations [22]. In this study, the egg white hydrolyzed with protease A and papain could change the surface charge of the egg white. In egg yolks, preserved egg whites and chickpea protein isolates, changes in protein charge after protease hydrolysis were also found, which are related to changes in the number of exposed ionizable amino groups and carboxyl groups exposed on the protein surface [23,24,25]. 

Colloids with high zeta potential (negative or positive) are electrically stabilized while colloids with low zeta potentials tend to coagulate or flocculate. Therefore, SLE + 1% EWH optimizes the foaming ability and foam stability. 

**Figure 1:**
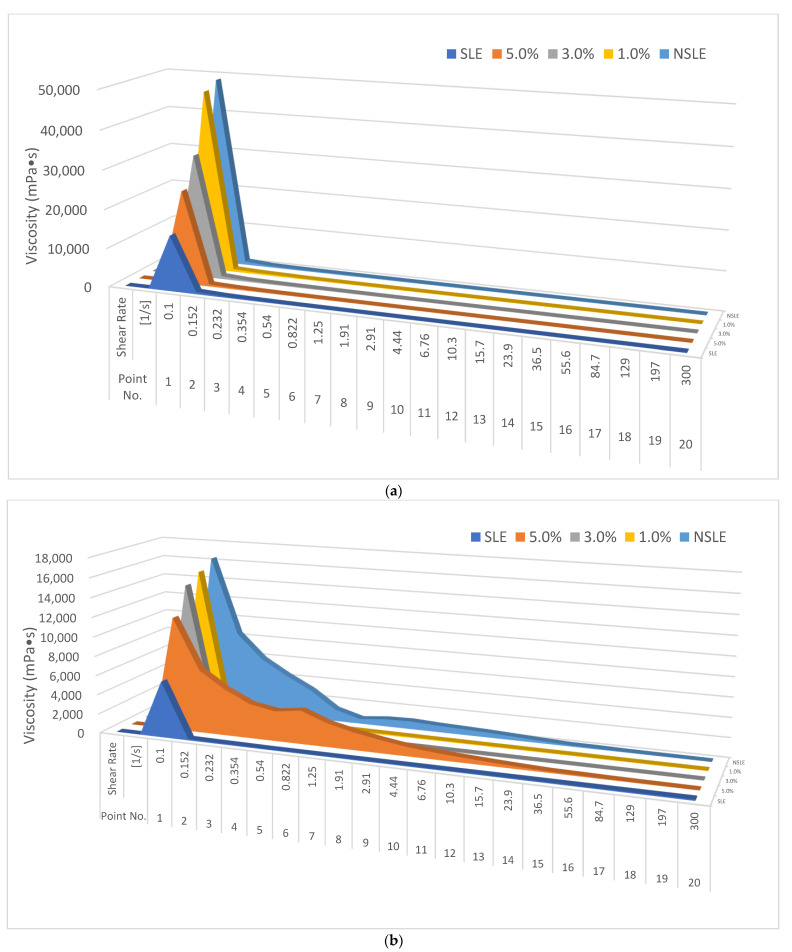
Viscosity of non-sterilized liquid egg white (NSLE), sterilized liquid egg white (SLE), and SLE supplemented with 1%, 3%, or 5% egg white hydrolysate (EWH) on Day 0 (**a**) and Day 7 (**b**).

The rheology (shear rate between 0.1 and 300 s^−1^) of the samples was determined to obtain the relationship between the viscosity and stability [26]. The liquid eggs with EWH underwent changes during storage that affected the EWH addition effect; therefore, the samples from Day 0 and Day 7 were used to determine the rheological properties. The rheological properties are discussed based on a 0.1 s^−1^ shear rate, because the error value was large. As shown in Figure 1a,b, the NSLE group had the highest viscosity on Day 0 and Day 7, whereas the SLE group had the lowest; the viscosity of SLE with different EWH additions also increased. The viscosity decreased with time and was not affected by EWH addition, as indicated in Table 1. Although EWH addition improved the stability of SLE, the effect decreased as the EWH concentration increased.

**Figure 2:**
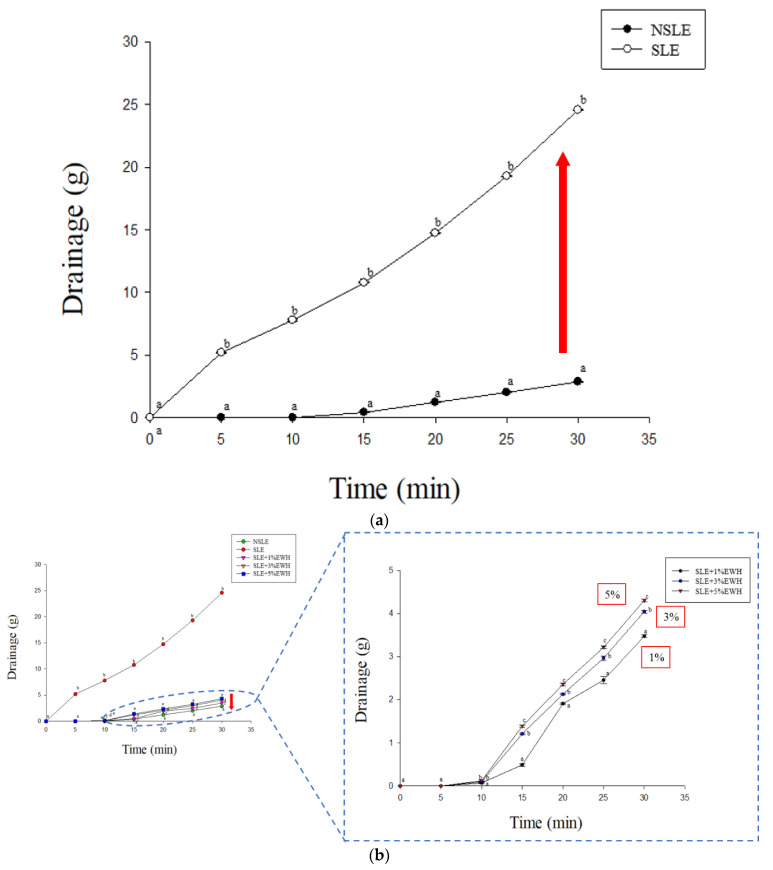
Drainage of foam made by (**a**) non-sterilized liquid egg white (NSLE) and sterilized liquid egg white (SLE), (**b**) NSLE, SLE, and SLE supplemented with 1%, 3%, or 5% egg white hydrolysate (EWH). ^a–c^ *p* < 0.05, significantly different from other groups.

Viscosity is a decisive factor in foam stability [19]; foam can be stabilized by increasing the viscosity of egg whites, whereas weight loss and stability have an inversely proportional relationship [27]. As shown in Figure 2a, SLE lost more water content than the NSLE group; SLE with EWH underwent lesser water loss than the SLE group, as shown in Figure 2b. Egg white with 1% EWH had the best stability (Figure 2b). These results prove that liquid egg has weaker stability after pasteurization, and EWH improves the stability; a 1% EWH addition conferred the best stability. A further increase in the EWH concentration lowered the stability of the liquid eggs.

### 3.3. Analysis of Foaming Characteristics

Based on previous studies, the foaming properties of whipped samples could be tested by physical analysis (OR, air phase, and foam density); a higher OR and air phase indicate lower density [14]. As shown in Table 2, the SLE group had lower OR and air phase values than the NSLE group but higher density. This implied that pasteurization of liquid eggs affected the foaming properties; nevertheless, the OR and air phase values were higher after EWH was added and increased with the concentration, but the density did not significantly vary. This showed that EWH enhanced the foaming properties of liquid eggs, and the effect improved with an increase in the EWH concentration (5% EWH induced the best foaming properties).

According to the literature, foam is defined as a two-phase system in which a continuous phase (such as water) is coated with a dispersed phase (such as air) [9,28]. From a structural point of view, foam is filled with a large number of bubbles and has a large volume [29]; the enlargement of bubbles represents the start of foaming and indicates better foaming properties [30]. Foaming properties can be observed using the bubbles formed and the rising height of the baked cake; larger bubbles and higher cake rise indicate better foaming properties [31]. In this study, we determined the foaming properties by baking honey cake, a type of sponge cake. Sponge cakes exhibit the least affected factors among baked cakes, and the main ingredients are eggs, sugar, and flour.

**Figure 3:**
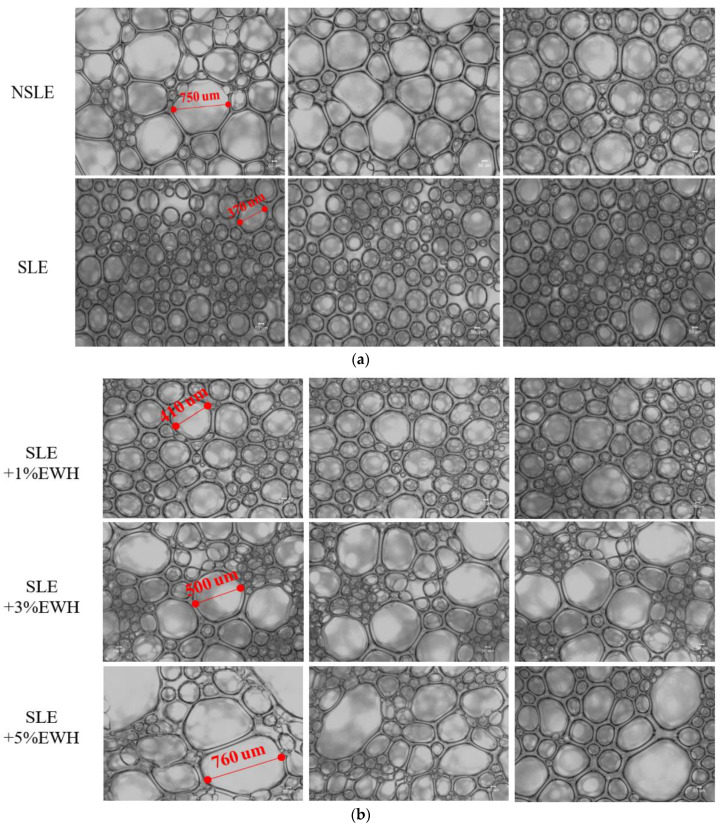
Micrographs (50×) of foam made with (**a**) non-sterilized liquid egg white (NSLE) and sterilized liquid egg white (SLE) and (**b**) SLE supplemented with 1%, 3%, or 5% egg white hydrolysate (EWH).

**Figure 4:**
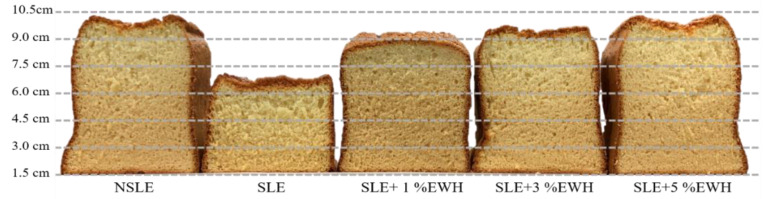
Cross-sectional heights of cakes made with non-sterilized liquid egg white (NSLE), sterilized liquid egg white (SLE), and SLE supplemented with 1%, 3%, or 5% egg white hydrolysate (EWH).

As shown in Figure 3a, the SLE group had a smaller average bubble size than the NSLE group, and according to Figure 4, the cake made using the SLE sample had a lower rising height than that made with the NSLE group. These results and those shown in Table 2 prove that pasteurization weakens the foaming properties of eggs. Moreover, SLE with different concentrations of EWH had significantly larger foam bubbles than the SLE group, irrespective of whether they were whipped or baked (Figure 3a,b). As shown in Figure 4, the rising height was also higher than that in the SLE group.

These results are similar to those in Table 2, that is, EWH improved the foaming properties, and the effect improved with an increase in the EWH concentration (5% EWH conferred the best foaming properties). According to previous studies, foam stability refers to the bubble size and spreading; hence, it is also a key factor in maintaining the structure of cakes [32]. If the bubbles are large and spread evenly, the stability is good; however, if the bubbles are not spread evenly, they would diminish easily. Therefore, Figure 3 indicates that 5% EWH conferred the best foaming properties, but the bubbles did not spread evenly and caused the cake to collapse; however, with 1% EWH, the cake volume was maintained, and the stability was good.

### 3.4. Organoleptic Analysis

Considering effective use in the baking industry and consumer preferences, this study used a nine-point hedonic scale test to determine consumer acceptance. The properties tested were appearance, smell, sweetness, bitterness, texture, and overall acceptance. This study included bitterness acceptance because there was a possibility of producing bitterness peptides, such as lysine with hydrophobic groups, valine, leucine, proline, phenylalanine, tyrosine, isoleucine, and tryptophan, after enzymatic hydrolyzation [15,33]. However, higher bitterness scores in this test indicated weaker bitter flavor. As shown in Table 3, SLE had a lower score than the NSLE group, implying that sterilized eggs were not acceptable in baking and food processing industries; however, cake made from SLE with EWH was accepted by consumers (1% EWH content received the best acceptance score among the EWH addition group).

### 3.5. Color Analysis

**Figure 5:**
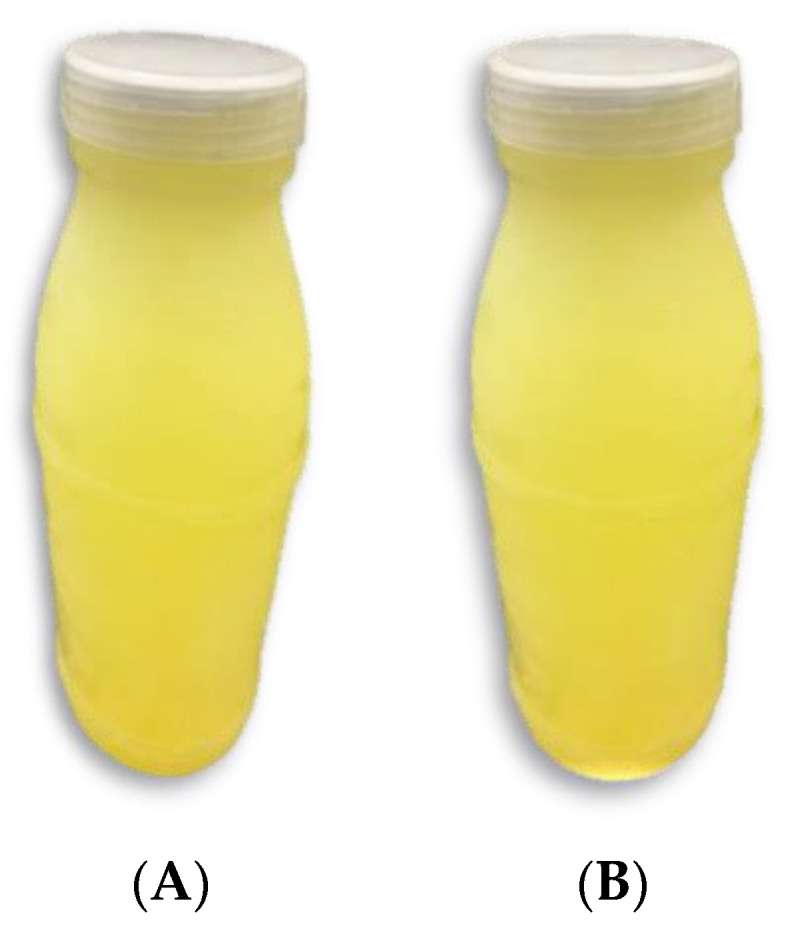
Commercially available sterilized liquid egg white (**A**) and sterilized liquid egg white supplemented with 1% egg white hydrolysate (**B**).

In this study, EWH was added as one of the baking ingredients, and it caused color changes in the product. Hence, the color of the baked cake was analyzed to ensure acceptability. Liquid eggs with EWH and baked cakes with EWH did not show significant changes in color (Figure 5). As indicated in Table 4 and Table 5, EWH addition decreased the lightness of the samples, but this could not be identified by naked eye in organoleptic analysis. Therefore, the addition of EWH did not affect consumer acceptance.

## 4. Conclusions

According to the literature, the highest DH of EWH is 25% [15]. EWH in this study had a 6.19% protein content and attained a DH of 30.11% upon enzyme hydrolyzation (protease A and papain). The negative charge potential value, surface tension, viscosity, and weight loss analysis indicated that EWH added to liquid eggs improved the foaming properties; 5% EWH addition conferred the best results. However, as the bubbles formed did not spread evenly, the baked cake collapsed. Nevertheless, 1% EWH could support the cake structure, and the appearance was acceptable to consumers. Therefore, to ensure its efficient use in the baking industry and considering the cost and stability, 1% EWH was chosen as the best concentration. It is hoped that this can overcome the limitations on the use of sterilized liquid eggs in the baking industry.

## Figures and Tables

**Table 1:** Zeta potential, surface tension, and viscosity of non-sterilized liquid egg white (NSLE), sterilized liquid egg white (SLE), and SLE supplemented with 1%, 3%, or 5% egg white hydrolysate (EWH).

Sample *	Zeta Potential (mV)	Surface Tension (erg/cm^2^)	Viscosity (mPa·s)
NSLE	−14.91 ± 0.16 ^a^	66.24 ± 0.48 ^a^	7356.67 ± 172.14 ^a^
SLE	−0.93 ± 0.11 ^e^	56.64 ± 0.48 ^e^	4056.67 ± 100.17 ^e^
SLE + 1% EWH	−12.65 ± 0.24 ^b^	63.52 ± 0.28 ^b^	6093.33 ± 41.63 ^b^
SLE + 3% EWH	−8.55 ± 0.12 ^c^	62.40 ± 0.48 ^c^	5580.00 ± 95.39 ^c^
SLE + 5% EWH	−4.16 ± 0.04 ^d^	61.12 ± 0.48 ^d^	5320.00 ± 65.57 ^d^

* Values are expressed as means ± standard deviations, and there is a significant difference between the averages indicated by different letters in the same column (*p* < 0.05).

**Table 2:** Physical analysis of foam made by non-sterilized liquid egg white (NSLE), sterilized liquid egg white (SLE), and SLE supplemented with 1%, 3%, or 5% egg white hydrolysate (EWH).

Sample *	Overrun (%)	Air Phase (%)	Foam Density (g/cm^3^)
NSLE	621.33 ± 0.10 ^a^	0.861 ± 0.00 ^a^	0.1283 ± 0.00 ^a^
SLE	575.51 ± 0.15 ^d^	0.852 ± 0.00 ^b^	0.1373 ± 0.00 ^b^
SLE + 1% EWH	600.50 ± 0.19 ^c^	0.857 ± 0.00 ^a^	0.1358 ± 0.00 ^b^
SLE + 3% EWH	612.50 ± 0.46 ^b^	0.860 ± 0.00 ^a^	0.1351 ± 0.00 ^b^
SLE + 5% EWH	625.30 ± 0.15 ^a^	0.862 ± 0.00 ^a^	0.1345 ± 0.00 ^b^

* Values are expressed as means ± standard deviations, and there is a significant difference between the averages indicated by different letters in the same column (*p* < 0.05).

**Table 3:** Organoleptic analysis of cake made with non-sterilized liquid egg white (NSLE), sterilized liquid egg white (SLE), and SLE supplemented with 1%, 3%, or 5% egg white hydrolysate (EWH).

Sample *	Appearance Acceptability	Aroma Acceptability	Sweetness Acceptability	Bitterness Acceptability	Texture Acceptability	Overall Acceptability
NSLE	6.38 ± 2.24 ^a^	6.40 ± 1.92 ^a^	6.50 ± 1.73 ^a^	8.20 ± 1.40 ^a^	6.63 ± 1.59 ^a^	6.75 ± 1.77 ^a^
SLE	5.83 ± 1.43 ^d^	5.88 ± 1.75 ^c^	5.88 ± 1.87 ^c^	7.85 ± 1.63 ^c^	5.58 ± 1.61 ^c^	6.08 ± 1.56 ^e^
SLE + 1% EWH	6.37 ± 1.82 ^a^	6.40 ± 2.02 ^a^	6.50 ± 1.50 ^a^	8.10 ± 1.74 ^b^	6.35 ± 1.66 ^b^	6.70 ± 1.45 ^b^
SLE + 3% EWH	6.28 ± 1.90 ^b^	6.38 ± 1.50 ^a^	6.70 ± 1.45 ^b^	8.08 ± 1.47 ^b^	6.35 ± 1.60 ^b^	6.20 ± 1.70 _d_
SLE + 5% EWH	6.20 ± 2.24 ^c^	6.31 ± 1.58 ^b^	6.69 ± 1.54 ^b^	8.06 ± 1.98 ^b^	6.60 ± 1.78 ^a^	6.40 ± 1.82 ^c^

* Values are expressed as means ± standard deviations, and ^a–e^ there is a significant difference between the averages indicated by different letters in the same column (*p* < 0.05). A total of 20 people carried out organoleptic analysis.

**Table 4:** Color analysis of non-sterilized liquid egg white (NSLE), sterilized liquid egg white (SLE), and SLE supplemented with 1%, 3%, or 5% egg white hydrolysate (EWH).

Sample *	L	a	b	ΔE
NSLE	87.46 ± 0.41 ^a^	−1.10 ± 0.19 ^a^	16.61 ± 0.15 ^a^	0.44 ± 0.39 ^a^
SLE	86.31 ± 0.34 ^b^	−1.07 ± 0.08 ^a^	17.24 ± 0.39 ^a^	1.00 ± 0.53 ^a^
SLE + 1% EWH	80.99 ± 0.34 ^c^	0.68 ± 0.48 ^b^	22.40 ± 1.38 ^b^	9.33 ± 1.37 ^b^
SLE + 3% EWH	79.51 ± 0.26 ^d^	0.89 ± 0.31 ^b,c^	23.08 ± 0.60 ^b^	9.92 ± 1.85 ^b^
SLE + 5% EWH	77.21 ± 0.17 ^e^	1.31 ± 0.06 ^c^	23.69 ± 0.05 ^b^	12.10 ± 0.15 ^c^

* Values are expressed as means ± standard deviations, and there is a significant difference between the averages indicated by different letters in the same column (*p* < 0.05). L: black and white/+ whiter–blackish; a: reddish green/+ reddish–greenish; b: yellowish blue/+ yellowish–blueish.

**Table 5:** Color analysis of cake made with non-sterilized liquid egg white (NSLE), sterilized liquid egg white (SLE), and SLE supplemented with 1%, 3%, or 5% egg white hydrolysate (EWH).

Cake Sample *	L	a	b	ΔE
NSLE	70.45 ± 0.46 ^a^	3.01 ± 0.17 ^a^	24.56 ± 0.21 ^a^	4.46 ± 0.26 ^a^
SLE	68.66 ± 0.26 ^b^	3.94 ± 0.75 ^a^	25.58 ± 0.90 ^a^	2.58 ± 0.95 ^b^
SLE + 1% EWH	67.73 ± 0.21 ^c^	4.83 ± 0.17 ^b^	26.99 ± 0.54 ^b^	0.63 ± 0.42 ^d^
SLE + 3% EWH	66.39 ± 0.33 ^d^	4.64 ± 0.56 ^b^	26.45 ± 0.94 ^b^	1.48 ± 0.60 ^c^
SLE + 5% EWH	65.12 ± 0.10 ^e^	4.65 ± 1.19 ^b^	26.78 ± 1.19 ^b^	2.65 ± 0.28 ^b^

* Values are expressed as means ± standard deviations, and there is a significant difference between the averages indicated by different letters in the same column (*p* < 0.05). L: black and white/+ whiter–blackish; a: reddish green/+ reddish–greenish; b: yellowish blue/+ yellowish–blueish.

## References

[1] Pande V.V., Gole V.C., McWhorter A.R., Abraham S., Chousalkar K.K. (2015). Antimicrobial resistance of non-typhoidal Salmonella isolates from egg layer flocks and egg shells. Int. J. Food Microbiol..

[2] Lechevalier V., Guérin-Dubiard C., Anton M., Beaumal V., Briand E.D., Gillard A., LeGouar Y., Musikaphun N., Tanguy G., Pasco M. (2017). Pasteurisation of liquid whole egg: Optimal heat treatments in relation to its functional, nutritional and allergenic properties. J. Food Eng..

[3] Hegg P.-O., Löfqvist B. (1974). The protective effect of small amounts of anionic detergents on the thermal aggregation of crude ovalbumin. J. Food Sci..

[4] Lomakina K., Míková K. (2006). A study of the factors affecting the foaming properties of egg white—A review. Czech J. Food Sci..

[5] Janssen H.J.L. (1971). Influence of pasteurisation, freezing and storage on properties of egg products made from eggs stored for 7 and for 28 days. J. Sci. Food Agric..

[6] Monfort S., Mañas P., Condón S., Raso J., Álvarez I. (2012). Physicochemical and functional properties of liquid whole egg treated by the application of Pulsed Electric Fields followed by heat in the presence of triethyl citrate. Food Res. Int..

[7] Robertson W.R., Muriana P.M. (2004). Reduction of Salmonella by two commercial egg white pasteurization methods. J. Food Prot..

[8] Romanoff A.L., Romanoff A. (1949). The Avian Egg. Bird-Banding.

[9] Wouters A.G.B., Rombouts I., Fierens E., Brijs K., Blecker C., Delcour J.A., Murray B.S. (2018). Foaming and air-water interfacial characteristics of solutions containing both gluten hydrolysate and egg white protein. Food Hydrocoll..

[10] Dabestani M., Yeganehzad S. (2019). Effect of Persian gum and Xanthan gum on foaming properties and stability of pasteurized fresh egg white foam. Food Hydrocoll..

[11] Li X., Yang Y., Murray B.S., Sarkar A. (2020). Combination of egg white protein and microgels to stabilize foams: Impact of processing treatments. J. Food Eng..

[12] Lahl W.J., Braun S.D. (1994). Enzymatic production of protein hydrolysates for food use. Food Technol. (Chicago).

[13] Nielsen P.M., Petersen D., Dambmann C. (2001). Improved method for determining food protein degree of hydrolysis. J. Food Sci..

[14] Li X., Li J., Chang C., Wang C., Zhang M., Su Y., Yang Y. (2019). Foaming characterization of fresh egg white proteins as a function of different proportions of egg yolk fractions. Food Hydrocoll..

[15] Horimoto Y., Lim L.T. (2017). Effects of different proteases on iron absorption property of egg white hydrolysates. Food Res. Int..

[16] Li J., Wang C., Li X., Su Y., Yang Y., Yu X. (2018). Effects of pH and NaCl on the physicochemical and interfacial properties of egg white/yolk. Food Biosci..

[17] Chalamaiah M., Esparza Y., Temelli F., Wu J. (2017). Physicochemical and functional properties of livetins fraction from hen egg yolk. Food Biosci..

[18] Xue Z., Worthen A., Qajar A., Robert I., Bryant S.L., Huh C., Prodanović M., Johnston K.P. (2016). Viscosity and stability of ultra-high internal phase CO_2_-in-water foams stabilized with surfactants and nanoparticles with or without polyelectrolytes. J. Colloid Interface Sci..

[19] Akhtar T.F., Ahmed R., Elgaddafi R., Shah S., Amani M. (2018). Rheological behavior of aqueous foams at high pressure. J. Pet. Sci. Eng..

[20] Lam A.C.Y., Warkentin T.D., Tyler R.T., Nickerson M.T. (2017). Physicochemical and functional properties of protein isolates obtained from several pea cultivars. Cereal Chem..

[21] Zhang J., Li Y. (2018). Study on the interaction between surfactin and alkaline protease in aqueous solution. Int. J. Biol. Macromol..

[22] Han Y., Huang X., Cao M., Wang Y. (2008). Micellization of surfactin and its effect on the aggregate conformation of amyloid β(1–40). J. Phys. Chem. B.

[23] Mokni Ghribi A., Maklouf Gafsi I., Sila A., Blecker C., Danthine S., Attia H., Bougatef A., Besbes S. (2015). Effects of enzymatic hydrolysis on conformational and functional properties of chickpea protein isolate. Food Chem..

[24] Ai M., Tang T., Zhou L., Ling Z., Guo S., Jiang A. (2019). Effects of different proteases on the emulsifying capacity, rheological and structure characteristics of preserved egg white hydrolysates. Food Hydrocoll..

[25] Gao Y., Li J., Chang C., Wang C., Yang Y., Su Y. (2019). Effect of enzymatic hydrolysis on heat stability and emulsifying properties of egg yolk. Food Hydrocoll..

[26] Giacintucci V., DiMattia C., Sacchetti G., Neri L., Pittia P. (2016). Role of olive oil phenolics in physical properties and stability of mayonnaise-like emulsions. Food Chem..

[27] Raikos V., Campbell L., Euston S.R. (2007). Effects of sucrose and sodium chloride on foaming properties of egg white proteins. Food Res. Int..

[28] Zmudziński D., Ptaszek P., Kruk J., Kaczmarczyk K., Roznowski W., Berski W., Ptaszek A., Grzesik M. (2014). The role of hydrocolloids in mechanical properties of fresh foams based on egg white proteins. J. Food Eng..

[29] Daugelaite D., Guillermic R.M., Scanlon M.G., Page J.H. (2016). Quantifying liquid drainage in egg-white sucrose foams by resistivity measurements. Colloids Surf. A Physicochem. Eng. Asp..

[30] Huang T., Tu Z.-C., Wang H., Shangguan X., Zhang L., Niu P., Sha X.-M. (2017). Promotion of foam properties of egg white protein by subcritical water pre-treatment and fish scales gelatin. Colloids Surf. A Physicochem. Eng. Asp..

[31] Noorlaila A., Hasanah H.N., Asmeda R., Yusoff A. (2020). The effects of xanthan gum and hydroxypropylmethylcellulose on physical properties of sponge cakes. J. Saudi Soc. Agric. Sci..

[32] Hill C., Eastoe J. (2017). Foams: From nature to industry. Adv. Colloid Interface Sci..

[33] Clemente A. (2000). Enzymatic protein hydrolysates in human nutrition. Trends Food Sci. Technol..

